# Protective Effects of Adipose Mesenchymal Stem Cell Secretome On Oxidative Stress-Induced Bisphenol-A in Isolated Rat Testes Mitochondria and Sperm Quality

**DOI:** 10.5935/1518-0557.20240089

**Published:** 2025

**Authors:** Maryam Zohour Soleimani, Layasadat Khorsandi, Yousef Asadi-Fard, Fatemeh Rezaei-Tazangi, Atefeh Ashtari

**Affiliations:** 1 Student Research Committee, Ahvaz Jundishapur University of Medical Sciences, Ahvaz, Iran; 2 Department of Anatomical Sciences, Faculty of Medicine, Ahvaz Jundishapur University of Medical Sciences, Ahvaz, Iran; 3 Cellular and Molecular Research Center, Medical Basic Sciences Research Institute, Ahvaz Jundishapur University of Medical Sciences, Ahvaz, Iran; 4 Department of Anatomy, School of Medicine, Arak University of Medical Sciences, Arak, Iran; 5 Department of Anatomy, School of Medicine, Fasa University of Medical Sciences, Fasa, Iran

**Keywords:** adipose-derived mesenchymal stem cell, secretome, Bisphenol-A, sperm, mitochondria

## Abstract

**Objective:**

This study aimed to explore the potential protective effects of adipose-derived mesenchymal stem cell secretome (ASE) on oxidative stress triggered by Bisphenol-A (BisA) exposure in testicular mitochondria and sperm quality of rats.

**Methods:**

Testicular tissue mitochondria and sperms were exposed to BisA (8 µM) and ASE (50 or 100 µg). ∆Ψm (mitochondrial membrane potential), reactive oxygen species (ROS) levels, antioxidant biomarkers, and sperm parameters were measured.

**Results:**

BisA elevated biomarkers of oxidative stress in mitochondria, while the levels of antioxidant activity and ∆Ψm decreased significantly. BisA harmed the morphology, survival rate, and mobility of the spermatozoids. ASE lowered malondialdehyde contents and ROS generation in the mitochondria, increased ∆Ψm, and reversed sperm quality.

**Conclusions:**

These data indicated that ASE effectively reduced BisA-induced damage to mitochondria and enhanced sperm quality by averting oxidative stress.

## INTRODUCTION

Infertility affects approximately 12% of the global population. The increased prevalence of infertility has been attributed to various factors such as a sedentary lifestyle, nutritional behavior, and environmental pollution (Bader *et al.*, 2019). Bisphenol-A (BisA) is a prominent plasticizer known for its exceptional cross-linking properties. Human exposure to BisA can occur through inhalation, dermal contact, and consumption of water and foods stored in plastic packaging (Liu *et al.*, 2022). Exposure to BisA has been associated with a range of health issues, particularly disorders affecting reproductive function.

BisA causes male reproductive toxicity, resulting in decreased sperm quality, reduced testosterone synthesis, and impaired supporting cell functions (Singh *et al.*, 2015; Tao *et al.*, 2019; Li *et al.*, 2021; Qi *et al.*, 2024). Different doses of BisA reduce sperm motility in fish, bovines, mice, and chickens (Hulak *et al.*, 2013; Lukacova *et al.*, 2015; Singh *et al.*, 2015). Chronic exposure to BisA decreased mouse sperm motility and impaired germ cell proliferation (Liu *et al.*, 2020). Furthermore, paternal exposure to BisA in CD-1 mice decreased total sperm counts and sperm motility among the offspring (Rahman *et al.*, 2015).

BisA induces cellular toxicity by interfering with the function and structure of mitochondria. Exposure to BisA results in oxidative stress and alterations in mitochondrial biogenesis, mitochondrial membrane potential (∆Ψm), and mitochondrial DNA (Wang *et al.*, 2019; Nayak *et al.*, 2022; Meng *et al.*, 2024; Qi *et al.*, 2024). BisA impairs the function of mitochondria by reducing ATP and lowering mitochondrial mass (Kaur *et al.*, 2018), which harms sperm motility. BisA reduces ∆Ψm in chicken sperms (Singh *et al.*, 2015) and elevates reactive oxygen species (ROS) levels in bovine and fish spermatozoa (Hulak *et al.*, 2013; Lukacova *et al.*, 2015). Accumulation of ROS may be responsible for causing BisA-induced germ cell toxicity (Wang *et al.*, 2019; Nayak *et al.*, 2022; Qi *et al.*, 2024). The physiological amount of ROS regulates normal spermatogenesis, while disruption of the oxidant-antioxidant system damages spermatogenesis and induces male infertility (Zhang *et al.*, 2023a). In the study of Khazaeel *et al.* (2022), BisA significantly declined normal sperm morphology, sperm count, motility, count, well as testosterone levels, CAT (catalase), and SOD (superoxide dismutase) activity. Also, BisA significantly increased the sperm anomalies, and MDA amount (Khazaeel *et al.*, 2022).

Mesenchymal stem cells (MSCs) for treating reproductive disorders have attracted the attention of recent researchers (Ahmed *et al.*, 2021; Zhang *et al.*, 2023b). MSCs derived from human amniotic membranes, bone marrow, and umbilical cords can improve chemical-induced testicular injuries (Qian *et al.*, 2020). Nafchi *et al.* (2024) reported that adipose-derived MSCs (ASCs) conditioned media improves human sperm count and motility. Adipose tissue, commonly known as fat, is abundant in the human body and can be easily harvested through minimally invasive procedures such as liposuction. This makes it a convenient and accessible source of stem cells for regenerative medicine and tissue engineering applications (Schneider *et al.*, 2017).

ASCs release hormones and growth factors into the surrounding environment, known as secretome (SE). SE derived from ASCs (ASE) has been reported to repair some degenerative disorders (Mehrabani *et al.*, 2015). SE has various advantages compared to cell-based therapies, such as being easily obtained, freeze-dried, packed, and transported (Khodayar *et al.*, 2022). ASE improved dog sperm quality after freezing and thawing (Qamar *et al.*, 2020). The co-culture of ASE improved human sperm vacuolization and DNA fragmentation (Bader *et al.*, 2019). Moreover, the antioxidant properties of ASCs and ASE have been reported (Kim *et al.*, 2008; Tofiño-Vian *et al.*, 2018; Alonso-Alonso *et al.*, 2020). Microvesicles from ASCs suppress oxidative stress in Osteoarthritic chondrocytes (Tofiño-Vian *et al.*, 2018). Another study reported the antioxidant property of ASCs in damaged alveolar epithelial cells (Peñuelas *et al.*, 2013).

Since the accumulation of ROS is the main mechanism of BisA-mediated mitochondrial dysfunction (Gassman, 2017; Nayak *et al.*, 2022), targeting the oxidative stress pathway may reverse the toxic effects of BisA on male germ cells. Due to the antioxidant and other beneficial impacts of ASE, this study aimed to investigate the protective effect of ASE against BSA-induced mitochondrial oxidative stress and sperm quality in rats.

## MATERIALS AND METHODS

### Study design (Figure 1)

Fifteen male Wistar rats (200-250 g) contributed their sperm and mitochondria to the study. The current study was approved by the Animal Research Ethics Committee (IR.AJUMS.ABHC.REC.1399.018).

**Figure 1:**
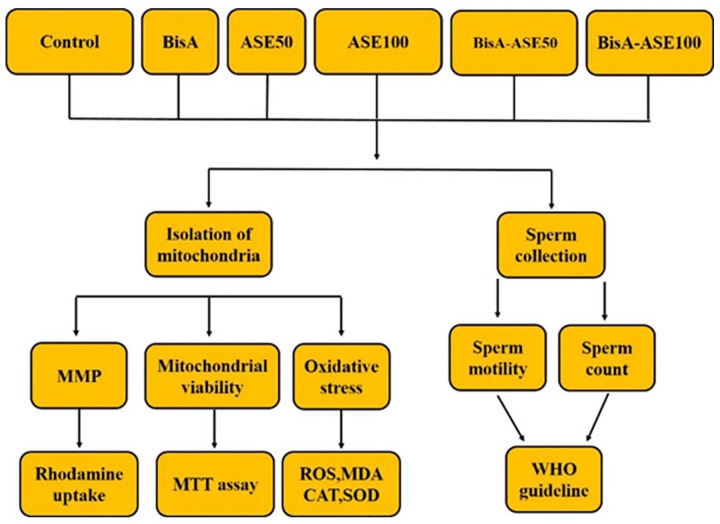
Schematic illustration detailing the experimental design of the study.

The epididymis sperm were separated and classified into the groups listed below:

Control. Given only Ham’s F-10 media for four hours. BisA. Received BisA (0.8 µM) for two hours

ASE50+ BisA. Received 50 µg of ASE, respectively, for two hours before BisA.

ASE100+ BisA. Received 100 µg of ASE, respectively, for two hours before BisA.

ASE100. Received 100 µg ASE for four hours

ASE or BisA treatment was applied to 5×10^6^ sperm/mL within each group. Because the untreated sperm expired after 4 hours, treatment with ASE or BisA was limited to a total of 4 hours. BisA (Sigma) was dissolved in 0.1% DMSO and mixed with Ham’s F-10 media. Concentration levels of BisA were determined using the results from the MTT test (Table 1).

**Table 1:** The impact of BisA on spermatozoa viability (Mean±SD; n=6).

Concentrations	1 hour	2 hours
0 (Control)	100.00±0.00	100.00±0.00
0.1 &mu;M	97.8±2.55	96.8±3.19
0.2 &mu;M	93.2±4.49	85.8±4.38^*^
0.4 &mu;M	75.4±5.47^*^	66.78±5.18^**^
0.8 &mu;M	62.7±5.18^**^	49.9±3.82^***^
1 &mu;M	56.1±3.39^**^	35.2±3.11^***^

* *p*<0.05,

** *p*<0.01,

*** *p*<0.001;

* comparison with control.

### Secretome Preparation

The characterized ASCs were obtained from the Royan Institute in Tehran, Iran. In short, the ASCs (1 × 105 cells/ well) were seeded onto 6-well plates and incubated in complete media. When reaching 80% confluence, the cells were washed with Hanks’ solution and cultured in serum-free media containing low glucose DMEM (Gibco, USA), 100 U/ ml penicillin/streptomycin (Sigma, USA), 2mM L-glutamine (Sigma, USA), and 10% AdvanceSTEM Stem Cell Growth (GE Life Sciences, USA) for three days. After collection, the conditioned media was subjected to centrifugation at 800. g for ten minutes to remove cell debris. The conditioned medium underwent a second round of centrifugation using an Amicon Ultra-15 centrifugal filter (Sigma, USA) for two hours. The protein concentrations in the supernatants were quantified using a Bradford assay and stored at -70°C.

### Mitochondria Isolation

The testicular tissues were chopped up and placed in a liquid mixture containing fat-free BSA (0.1%), sucrose (250mM), HEPES-KOH (5mM), EGTA (0.2mM), and EDTA (0.1mM). The sample, after the homogenization process, underwent four rounds of centrifugation in an isolating buffer containing 1.0 M sucrose, 0.5 M MgCl2, 0.1 M KCl, 0.1 M EGTA, 0.1 M K2HPO4, 1.0 M mannitol, 0.1 M MOPS, 0.5 M HEPES, 10% BSA, 0.5 M glutamate, and 1.0 M succinate at a temperature of 4°C to separate the mitochondrial fractions: once at 3,000· g (for 10 min) and three times at 10,000·g (for 7 min each). The protein concentrations in the supernatants were quantified using a Bradford assay and stored at -70°C.

### Sperm Parameters

The epididymis was dissected and placed in a 1% trisodium citrate solution (1 mL) for eight minutes. Eight mL of the trisodium citrate solution were added and mixed for one minute. The spermatozoa suspension was diluted in 10% formalin at a 1:1 ratio. Spermatozoa were counted using a Neubauer hemocytometer. A 100 µL sperm suspension was placed on a glass slide for morphological observation. Morphological evaluation was performed on 100 spermatozoa in each mouse. Sperm mobility was assessed according to the protocols established by the World Health Organization. Suspended sperm (10 µL) was poured into the semen analysis chamber. Six fields were evaluated for the motility rate of at least 200 sperm for each specimen.

### Determining antioxidant levels, MDA content, and ROS formation

The mitochondria were incubated in DCFH-DA (10 µM; Sigma) along with Hanks buffered salt solution (100 µL) for 25 minutes. The ROS levels were determined using a spectrofluorometer with an excitation wavelength of 490 nm and an emission wavelength of 570 nm. Levels of superoxide dismutase (SOD), catalase (CAT), as well as malondialdehyde (MDA) were examined using commercially available kits from ZellBio Company.

### Mitochondrial membrane potential (∆Ψm) evaluation

The mixture of Rhodamine 123 (10 µM) and mitochondrial fractions was prepared. The fluorescence was recorded using a spectrophotometer (LS50B, USA) with an excitation wavelength of 490 nm and an emission wavelength of 535 nm.

### Statistical Analysis

In this study, the data were analyzed using a one-way analysis of variance in SPSS (version 21.0), with post-hoc pairwise comparisons. Statistical significance was determined for the *p*-values less than 0.05.

## RESULTS

### Sperm Quality

**Figure 2:**
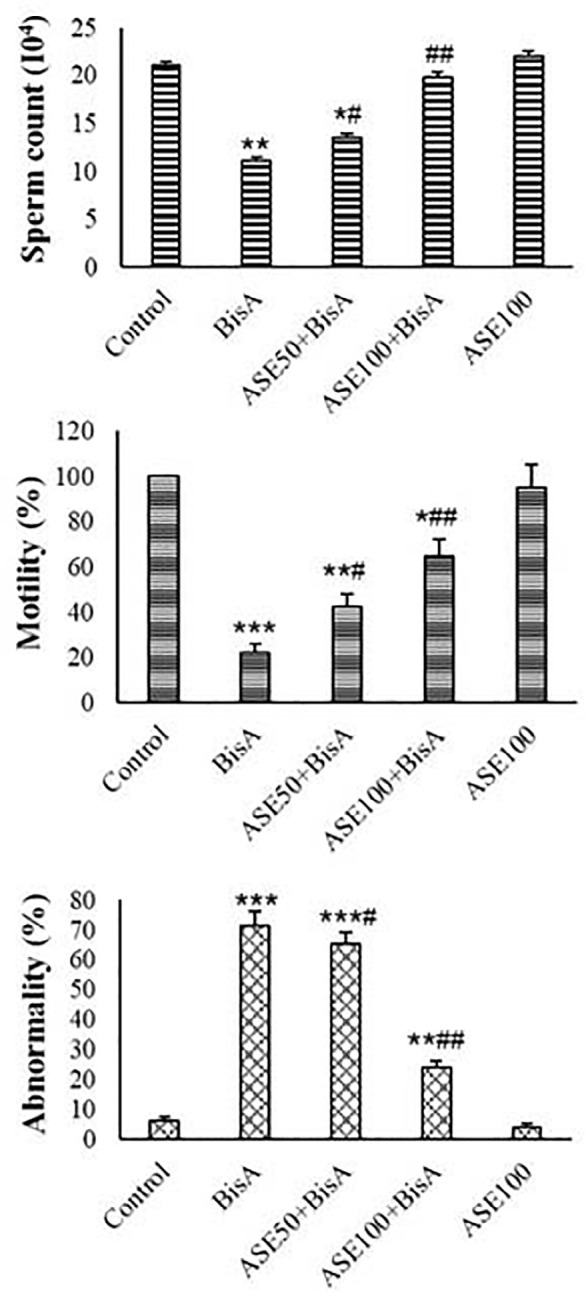
Total rat sperm motility (%), abnormality (%), and count in the various groups (mean±SD, n=5). * and # indicate comparisons against control and BisA-treated groups, respectively.

ASE (100 µg) had no significant impact on the number, motility, and typical morphology of the sperms. In the treatment with BisA, a noticeable reduction in sperm motility and numbers (*p*<0.01) was observed compared to the control. Bis-A significantly increased the percentage of immotile sperms compared to the control (*p*<0.01). ASE pretreatment, concentration-dependently, caused a significant reduction in the number of immotile sperm while enhancing overall sperm motility in the BisA treatment (Figure 2).

The level of abnormality in sperm exposed to BisA was significantly more than the control (*p*<0.01). Pre-exposure to ASE resulted in a dose-dependent decrease in sperm abnormalities compared to the BisA group. ASE at the concentration of 100 µg had a more positive impact on sperm quality than the 50 µg (Figure 2).

### Antioxidant levels, MDA content, and ROS formation

**Figure 3:**
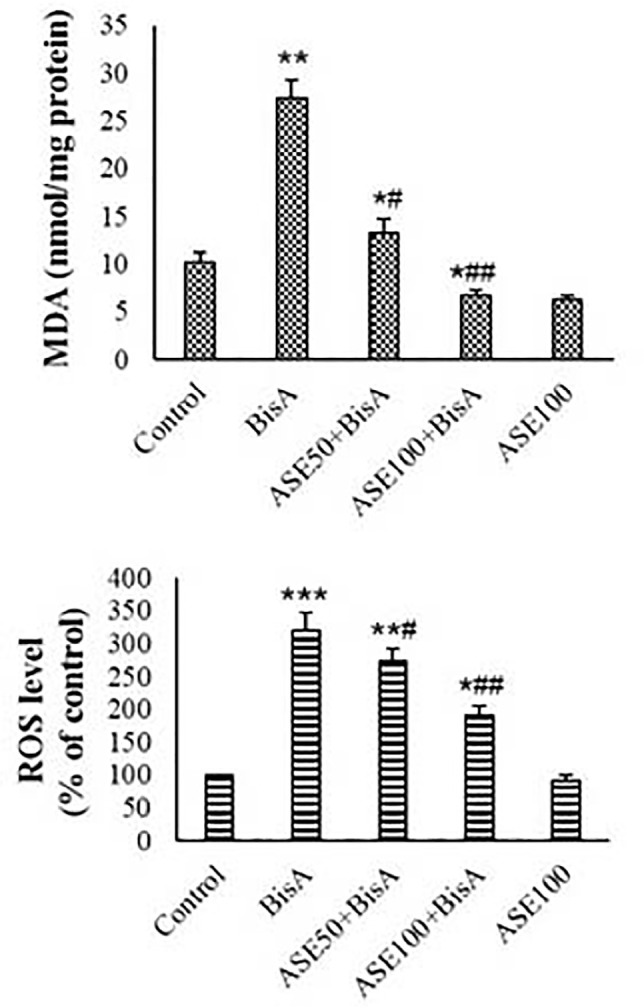
ROS and MDA levels in the mitochondrial fractions (mean±SD, n=5). * and # indicate comparisons with untreated control and BisA-treated groups, respectively.

**Figure 4:**
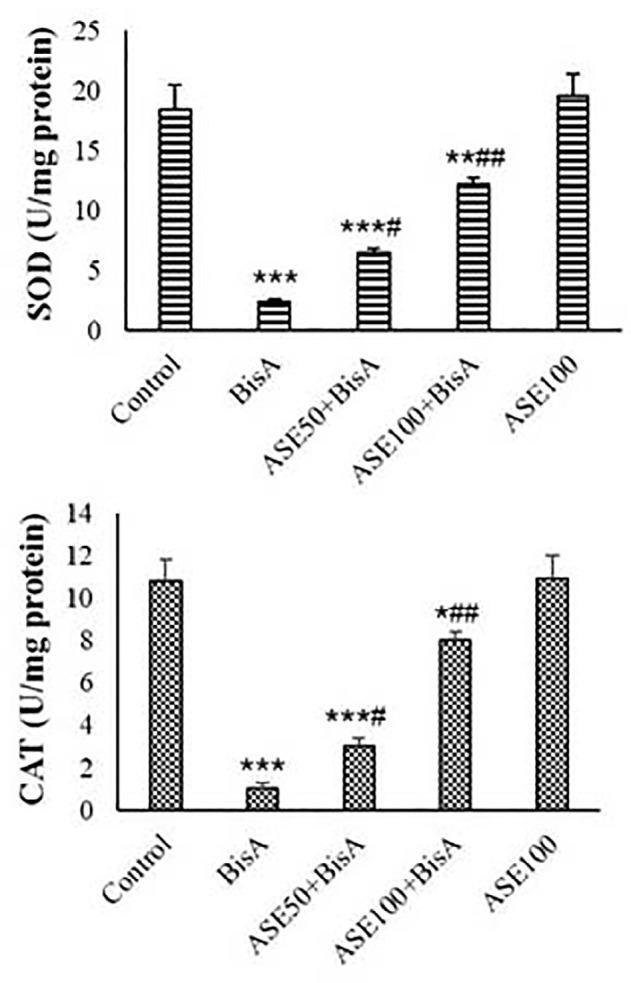
CAT and SOD levels in the mitochondrial fractions (mean±SD, n=5). * and # indicate comparisons with untreated control and BisA-treated groups, respectively.

No significantly lower levels of MDA and ROS were observed in the groups that received ASE (100 µg) compared to the control. Levels of ROS and MDA showed a significant increase in the BisA group compared to the control (*p*<0.01). ASE concentration-dependently reduced MDA and ROS levels in the BisA-exposed mitochondria (Figure 3). The mitochondria exposed to only ASE (100 µg) exhibited no significantly higher levels of CAT and SOD activity than the untreated groups. Treatment with BisA resulted in decreased activity of CAT and SOD enzymes in the isolated mitochondria compared to the control (*p*<0.001). In a concentration-dependent way, ASE reversed the CAT and SOD activity of the mitochondria. ASE at the concentration of 100 µg had more effect on the reducing MDA and ROS levels and increased activity of CAT and SOD enzymes compared to the 50 µg (Figure 4).

### ∆Ψm Assay

**Figure 5:**
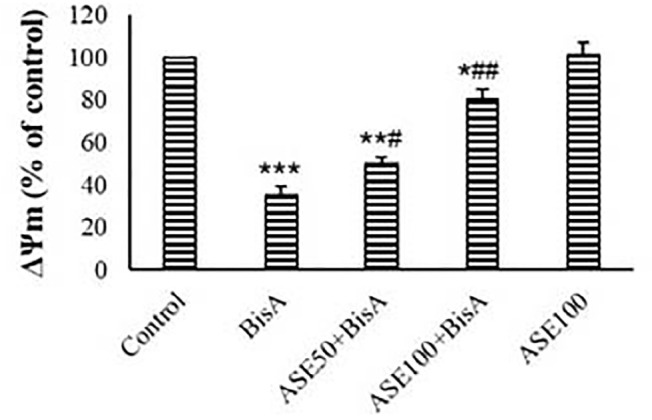
∆Ψm measurement in different groups (mean±SD, n=5). * and # indicate comparisons against untreated control and BisA-treated groups, respectively.

∆Ψm was not significantly changed in ASE (100 µg)-treated cells compared to the control. There was a significant decrease in ∆Ψm within the BisA group compared to the control group (*p*<0.01) (Figure 5). ASE could dose-dependently reverse the ∆Ψm of BisA-exposed mitochondria. 100 µg of ASE showed more impact on the ∆Ψm than 50 µg (*p*<0.01).

## DISCUSSION

In this study, ASE could improve sperm quality, attenuate oxidative stress, and increase ∆Ψm of BisA-exposed sperm and testicular tissue mitochondria. BisA significantly decreased sperm motility, normal morphology, and sperm count. In parallel with our results, previous studies conducted in humans and animals revealed the negative impacts of BisA on sperm parameters (Knez *et al.*, 2014; Kotwicka *et al.*, 2016; Cariati *et al.*, 2019; Liu *et al.*, 2020; Khazaeel *et al.*, 2022; Lü *et al.*, 2024). An increased number of abnormal spermatozoa after 4 hours of BisA exposure was reported by Nguyen *et al.* (2022). A meta-analysis study revealed that BisA impairs sperm quality (Lü *et al.*, 2024).

In our study, the decreased sperm quality caused by Bisphenol A (BisA) was associated with increased MDA and ROS levels within the isolated testicular tissue mitochondria. The overproduction of ROS hurts spermatozoa, resulting in elevated MDA production and subsequent lipid peroxidation (Hsieh *et al.*, 2006; Wagner *et al.*, 2017).

Due to low cytoplasm, spermatozoa lack mechanisms for relieving oxidative damage (Agarwal *et al.*, 2014). Additionally, the rich polyunsaturated fatty acids of sperm membranes make them susceptible to oxidative damage through lipid peroxidation (de Lamirande & Gagnon, 1995; Alahmar, 2019; Rahman & Pang, 2019). Other studies have also demonstrated enhanced lipid peroxidation and ROS levels following exposure to BisA in spermatozoa (Rahman *et al.*, 2016; Gassman, 2017).

The ROS elevation levels were along with a decrease in ∆Ψm in the BisA group. Exposure to BisA can cause a reduction in ∆Ψm for human sperm, even at very low doses (Grami *et al.*, 2020). The regulation role of the ∆Ψm is crucial for maintaining mitochondrial function, structure, and metabolism. Additionally, ∆Ψm regulates ROS generation and removes damaged mitochondria (Zorov *et al.*, 2014). BisA alters ∆Ψm to promote mitochondrial dysfunction and negatively affects ATP levels in mouse spermatozoa (Kaur *et al.*, 2014; Jiang *et al.*, 2015; Rahman *et al.*, 2016; Shirani *et al.*, 2019).

The decreased sperm quality in the BisA group was along with a reduction in ∆Ψm. ∆Ψm has a direct positive impact on both the mobility and quantity of sperm cells (Madeja *et al.*, 2021). BisA-mediated mitochondrial dysfunction may stimulate the mitophagy pathway (Meng *et al.*, 2024), thus interfering with mitochondrial mass.

Moreover, Bisphenol A (BisA) may induce germ cell apoptosis leading to decreased sperm quality. The decreased ∆Ψm by BisA supports this hypothesis (Zhang *et al.*, 2019). The apoptotic impact of BisA on testicular cells has been reported in many studies (Zhang *et al.*, 2017, 2023a; Chen *et al.*, 2022). Increased germ cell apoptosis was observed during sperm development following postnatal exposure to BisA (Shaha *et al.*, 2010; Xie *et al.*, 2016).

Our research revealed that ASE improved sperm count, normal morphology rate, and motility of the rats treated with BisA, corroborating findings from previous studies (Bader *et al.*, 2019; Qamar *et al.*, 2020). Bader *et al.* (2019) demonstrated that ASE improves sperm motility and viability. ASE may enhance sperm quality by suppressing apoptosis. In addition, ASE might reduce levels of ROS by enhancing ∆Ψm, which could subsequently decrease germ cell apoptosis. The antiapoptotic impact of ASE has been reported in previous studies (Jiao *et al.*, 2021; Seo *et al.*, 2022). ASCs inhibited apoptosis caused by ionizing radiation in the testicular tissue (Cetinkaya-Un *et al.*, 2022). Jiao *et al.* (2021) reported that ASE reversed the ischemia-reperfusion-induced by decreasing the P53, Bax, Fas, FasL, and Bcl-2 expression.

In our study, the enhanced SOD and CAT levels indicate the antioxidant properties of ASE against BisA. The antioxidant effects of ASCs have also been demonstrated in dermal fibroblasts, degenerative diseases of the retina, and osteoarthritic chondrocytes (Kim *et al.*, 2008; Tofiño-Vian *et al.*, 2018; Alonso-Alonso *et al.*, 2020). Based on our findings, ASE exhibited a concentration-dependent reversal of ∆Ψm and ROS production. Therefore, ASE may protect testicular mitochondria by minimizing oxidative stress. This data aligns with existing research that shows the positive effects of ASE on mitochondrial function (Ma *et al.*, 2022).

The secretome derived from other sources of the MSCs has also been shown to have positive impacts on sperm parameters. Cai *et al.* (2021) reported that bone marrow-derived secretome improves spermatogenesis in mice with busulfan-induced azoospermia. Canine amniotic membrane-derived MSCs protected dog sperm in the freezing and thawing processes (Mahiddine *et al.*, 2020).

## CONCLUSION

In summary, ASE improved ∆Ψm and declined mitochondrial oxidative damages. ASE also enhanced rat sperm quality. It is suggested that ASE might ameliorate Bis-Ainduced mitochondrial injury and rat sperm quality impairment by inhibiting oxidative stress. Future studies are required to clarify the mechanism of ASE on spermatozoa and mitochondrial-related pathways such as mitophagy and apoptosis. This primary study stimulates the research to explore ASE’s benefits on male infertility disorders.
